# Development of healthy lifestyle consciousness index for gynecological cancer patients

**DOI:** 10.1007/s00520-022-07179-z

**Published:** 2022-06-08

**Authors:** Nozomi Higashiyama, Ken Yamaguchi, Yosuke Yamamoto, Akihiko Ueda, Yoshihide Inayama, Miho Egawa, Koji Yamanoi, Mana Taki, Masayo Ukita, Yuko Hosoe, Akihito Horie, Junzo Hamanishi, Masaki Mandai

**Affiliations:** 1 Department of Gynecology and Obstetrics, Graduate School of Medicine, Kyoto University, 54 Kawahara-cho, Shogoin, Sakyo-ku, Kyoto, 606-8507 Japan; 2 Department of Healthcare Epidemiology, School of Public Health, Graduate School of Medicine, Kyoto University, Yoshida-Konoe-cho, Sakyo-ku, Kyoto, 606-8501 Japan

**Keywords:** Healthy lifestyle, Gynecological neoplasms, Quality of life, Cancer

## Abstract

**Purpose:**

Healthy lifestyle is related to quality of life (QOL) after cancer diagnosis and prognosis. However, there are few reports on patients conscious of healthy lifestyle and patients requiring medical providers’ attention regarding healthy lifestyle. We aimed to develop a healthy lifestyle consciousness index (HLCI) for cancer patients and evaluated its validity in gynecological cancer patients.

**Methods:**

The HLCI was designed to assess degree of healthy lifestyle consciousness, including items regarding “diet,” “exercise,” “body weight,” and “sleep.” Exploratory factor analysis was performed for dimensionality of the scale; Cronbach’s alpha was calculated to assess internal-consistency reliability. For criterion-based validity, we calculated proportions of stage III/IV gynecological malignancies in those with categorized HLCI scores based on tertiles. Concurrent validity was evaluated between HLCI and other quality of life (QOL) scales including European Organization for Research and Treatment of Cancer QLQ-C30 in limited patients.

**Results:**

HLCI comprised five 10-point items (0–45); higher values implied improved healthy lifestyle consciousness. Data from 108 gynecological malignancy patients at Kyoto University Hospital were analyzed. The mean age of subjects was 55.8 years; 36.1% of them had uterine corpus cancer; 34.3% were at stage III/IV of gynecological malignancy. The factor analysis revealed HLCI was unidimensional; the reliability based on Cronbach’s alpha was satisfactory (0.88). The proportions of stage III/IV gynecological malignancies were 25.7%, 33.3%, and 44.4% in those with first (7–24 points), second (25–30 points), and third (31–46 points) tertiles of HLCI score, respectively. For patients with other QOL scales (*n* = 25), the mean scores of global health status of QLQ-C30 were 33.3, 50.0, and 83.3 for first, second, and third tertiles of HLCI score, respectively.

**Conclusion:**

HLCI was successfully validated; thus, patients with advanced stages or higher QOL might have strong consciousness regarding healthy lifestyle. HLCI may be useful in precision care for improved lifestyles and QOL.

## Introduction

A healthy lifestyle associated with adequate nutrition and exercise is related to the quality of life (QOL) after cancer diagnosis and prognosis [1–5]. Exercise is reported to significantly increase health-related QOL in several cancers, including gynecological malignancies [5]. Various interventions for lifestyle have also been reported, including telephone [6], e-mail [7], face-to-face intervention [8], and social cognitive theory [9]. However, it is difficult to implement intensive interventions using these resources for all cancer patients. Therefore, interventions of varying intensities are needed depending on the degree health consciousness of the patient. However, there is no index to measure the health consciousness of cancer patients. The identification of low consciousness regarding healthy lifestyles during cancer treatment can lead to precision healthcare for cancer patients.

We aimed to develop a healthy lifestyle consciousness index (HLCI) for cancer patients and to evaluate its reliability and validity in gynecological cancer patients.

## Methods

### Development of the HLCI

The items of the HLCI in gynecological cancer patients were generated by two gynecological oncologists and an epidemiologist. Based on a review of previous studies, all members discussed and generated item pools related to healthy lifestyles in cancer patients. After extracting items that could be useful in evaluating the degree of consciousness of healthy lifestyle, face validity of the questionnaire was checked by few patients. We asked them whether they felt any difficulty while answering the selected items.

### Study participants and ethical issues

Participants were recruited from patients who regularly visited Kyoto University Hospital owing to gynecological cancer. Patients who were literate in Japanese and were ≥ aged 20 years were included. The protocol for this study was approved by the Kyoto University Graduate School and Faculty of Medicine Ethics Committee (C1509). Written informed consent was obtained from all participants.

### Measurements

We collected data on HLCI and the following patient characteristics: age, types of cancer, primary or recurrence of the cancer, stage, and treatment status. Furthermore, quality of life (QOL) scales including the European Organization for Research and Treatment of Cancer (EORTC) QLQ-C30 [10] and Patient Health Questionnaire-9 (PHQ9) [11] were obtained from limited patients who agreed to be assessed by other QOL scales.

### Evaluation of the reliability and validity of HLCI

#### Factor structure

Factor analysis was performed to assess the dimensionality of HLCI. The data were analyzed to identify underlying components using exploratory factor analysis based on an iterated principal factor method. The number of components was determined by an eigenvalue > 1.

#### Reliability

The internal-consistency reliability of the scores on the HLCI was evaluated by calculating Cronbach’s alpha. Cronbach’s alpha > 0.7 was considered to indicate sufficient internal consistency.

#### Criterion-based validity

To assess criterion-related validity, we categorized participants into three groups using tertiles (T1, low tertile; T2, middle tertile; T3, high tertile) of the total scores if the uni-dimensionality of the scale was ascertained based on the results of factor analysis. We calculated the proportions of having clinical backgrounds including the stage III/IV of gynecological malignancies, recurrence of the cancer, and treatment status in those with categorized HLCI scores according to the tertiles; this was based on the hypothesis that patients with higher HLCI are likely to present higher proportions of stage III/IV, recurrent of cancer, and during-treatment status. To evaluate concurrent validity, mean scores of the global health status of EORTC and PHQ9 were presented for those with categorized HLCI scores according to the respective tertiles.

#### Statistical analysis

The patient characteristics were summarized using descriptive statistics. The Spearman test was used to understand the correlation between PRO and HLCI. *P*-value < 0.05 was considered statistically significant. The statistical software Python version 3.10 was used for statistical analyses.

## Results

### Healthy lifestyle consciousness index

The HLCI included five items. “Diet,” “exercise,” “body weight,” and “sleep” are the major components of lifestyle; thus, the HLCI consisted five questionnaires including these four items and a questionnaire on general health awareness. The following were the five questionnaires scaled from 0 to 9: (1) Do you live with consciousness of healthy lifestyle and habits? (2) Do you manage diets with consciousness of cancer in your daily life? (3) Do you exercise with consciousness of cancer in your daily life? (4) Do you manage your body weight with consciousness of cancer in daily life? (5) Do you manage to sleep with consciousness of cancer in your daily life? HLCI was calculated as the sum of the scores of the questionnaires (Table 1). HLCI was designed to evaluate the consciousness of patients regarding healthy lifestyle and not healthy lifestyle behaviors.

**Table 1:** Healthy lifestyle-consciousness index (HLCI)

Questions	Index scores
Do you live with consciousness of a healthy lifestyle and habits?	0 to 9
Do you manage diets with consciousness of cancer in your daily life?	0 to 9
Do you exercise with consciousness of cancer in your daily life?	0 to 9
Do you manage your body weight with consciousness of cancer in daily life?	0 to 9
Do you manage to sleep with consciousness of cancer in your daily life?	0 to 9
Healthy lifestyle-consciousness index (HLCI) = the sum all questionnaire scores	0 to 45

### Background of patients

In total, 108 patients were included in the present study. Patient backgrounds are summarized in Table 2. The mean age of the 108 patients was 55.8 years (standard deviation [SD]: ± 12.2 years). Thirty-three patients (30.6%) had uterine cervical cancer; 39 (36.1%) had uterine corpus cancer; 34 (31.5%) had ovarian, fallopian tube, and peritoneal cancer; one (0.9%) had double cancer including uterine corpus cancer and ovarian cancer; and one (0.9%) had vulvar cancer. Ninety patients (83.3%) were diagnosed with primary disease, whereas 18 patients (16.7%) had recurrent disease. Fifty-four patients (50.0%) were diagnosed with stage I, 17 (15.7%) with stage II, 24 (22.2%) with stage III, and 13 (12.0%) with stage IV disease. Thirty-one patients (28.7%) were before treatment, 45 patients (41.7%) were under treatment, and 32 patients (29.6%) were under follow-up after treatment.

**Table 2:** Backgrounds of study participants

Factors	Variable	Number of the cases	%
Age	30 s	8	7.4
	40 s	29	26.9
	50 s	30	27.8
	60 s	23	21.3
	More than 70	18	16.7
Types	Uterine cervical cancer	33	30.6
	Uterine corpus cancer	39	36.1
	Ovarian, fallopian tube, and peritoneal cancers	34	31.5
	Double cancer	1	0.9
	Vulvar cancer	1	0.9
Primary or recurrence	Primary	90	83.3
	Recurrence	18	16.7
Stage	I	54	50.0
	II	17	15.7
	III	24	22.2
	IV	13	12.0
Treatment status	Before treatment	31	28.7
	During treatment	45	41.7
	After treatment	32	29.6

### Structural validity of the HLCI

**Fig. 1:**
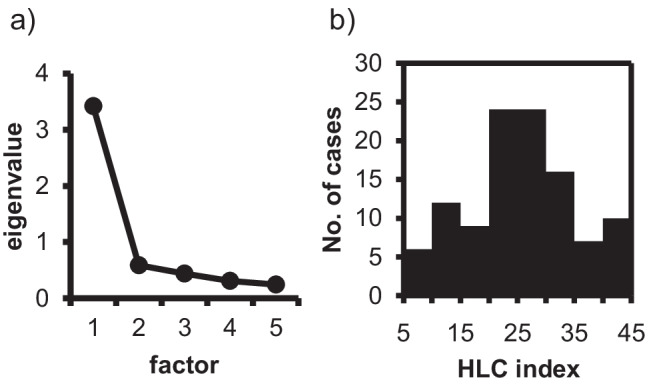
Exploratory factor analysis (**a**) and histogram of the number of cases in the healthy lifestyle-consciousness index (HLCI). HLCI has a uni-dimensional structure based on the pattern of eigenvalues and it decreases remarkably with the second factor and later factors (**a**). The HLCI was localized in Gaussian distribution (**b**)

The exploratory factor analysis showed that HLCI in gynecological cancer patients exhibited a unidimensional structure based on the pattern of eigenvalues; it decreased remarkably with the second factor and later factors (Fig. 1a). This implies that healthy lifestyle consciousness among gynecological cancer patients comprises a single concept of “healthy lifestyles.” Therefore, the total additional score of each questionnaire on the healthy lifestyle index in cancer patients was used in this study. The HLCI ranged from 0 to 45. The mean HLCI was 25.1 (SD: ± 9.3); the minimum and maximum HLCI in this study were 5 and 44, respectively. The distribution of HLCI score is shown in Fig. 1b, indicating no remarkable floor and ceiling effects in patients of the present study (Fig. 1b).

### Reliability of HLCI

The Cronbach alpha for all five questionnaires was 0.88, indicating high reliability.

### Criterion-based validity: relationship between HLCI and clinical status

**Fig. 2:**
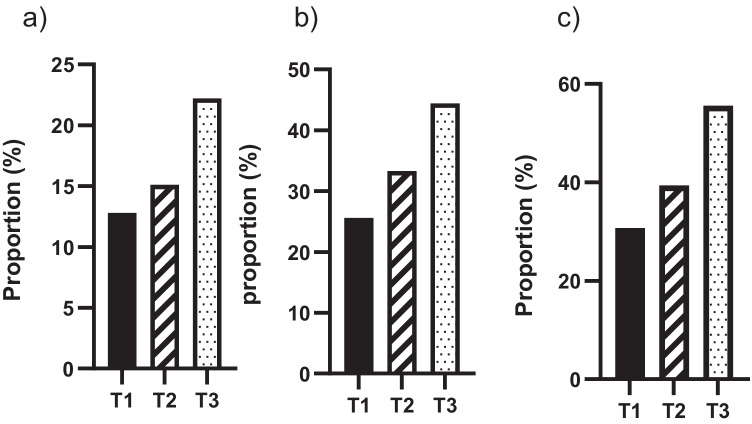
Criterion-related validity of HLCI in gynecologic cancer patients was compared using clinical backgrounds. Three tertiles (T1; low tertile, T2; middle tertile, T3; high tertile) based on the scores of HLCI were evaluated in patients with recurrence (**a**), stage III and IV (**b**), and during treatment (**c**)

To evaluate the criterion-related validity of HLCI, gynecological cancer patients were categorized using tertiles (T1, low tertile, *n* = 39; T2, middle tertile, *n* = 33; T3, high tertile, *n* = 36) based on the scores of HLCI and compared using clinical backgrounds. The proportion of patients with recurrent disease in T3 was higher than that in T2 and T1 (22.2%, 15.1%, and 12.8%, respectively; Fig. 2a). Similarly, the proportion of patients with stage III and stage IV disease in T3 was higher than that in T2 and T1 (44.4%, 33.3%, and 25.6%, respectively; Fig. 2b). Considering patients during treatment, the proportion was the highest in T3 followed by T2 and T1 (55.6%, 39.4%, and 30.7%, respectively; Fig. 2c).

### Concurrent validity: relationship between HLCI and other QOL scales

**Fig. 3:**
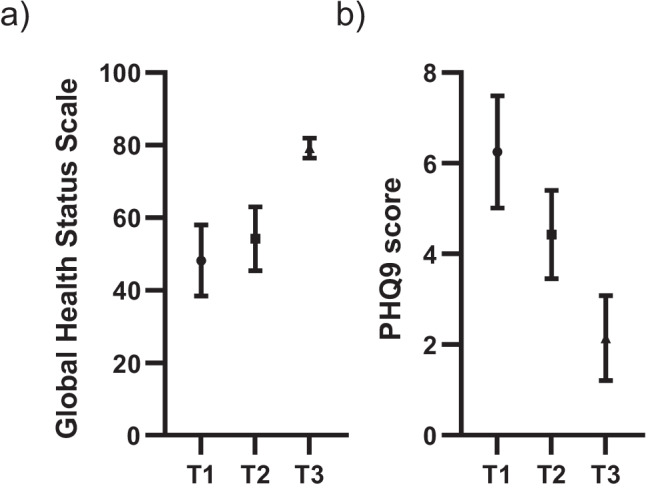
Concurrent validity of HLCI in gynecologic cancer patients was evaluated by comparing QOL. Three tertiles (T1; low tertile, T2; middle tertile, T3; high tertile) based on the scores of HLCI were evaluated in global health status from EORTC QLQ C-30 (**a**) and PHQ9 (**b**). Data represented are mean ± standard error

To evaluate the concurrent validity of HLCI, three groups categorized using tertiles based on the HLCI scores in gynecological cancer patients were compared in QOL scales comprising global health status of EORTC QLQ-C30 and PHQ9 (*n* = 25). The global health status, a measure of the overall QOL, was highest in T3 (mean value = 79.2) compared to T2 and T1 (mean values = 54.2 and 48.1, respectively; Fig. 3a). The global health status and HLCI were significantly correlated (Spearman *r* = 0.43, *p* = 0.03). The PHQ9 score, a measure of depressive symptoms, was highest in T1 (mean value = 6.3) compared to T2 and T3 (mean values = 4.4 and 2.1, respectively; Fig. 3b). The PHQ9 and HLCI were significantly correlated (Spearman *r* =  − 0.45, *p* = 0.04).

## Discussion

We developed the first HLCI for cancer patients and tested its reliability and validity in gynecological patients. The results showed that HLCI can be used to assess the consciousness of healthy life among cancer patients. Published reports showed the relationships between healthy lifestyles and QOL after cancer diagnosis and prognosis [1–5]. Self-management of lifestyle including exercise and diet reportedly improved the QOL of cancer patients and increased survival rates. Currently, there is no index to evaluate the consciousness regarding healthy lifestyle among cancer patients; thus, employing a clear external standard is difficult. However, there are few reports on the association between healthy lifestyle and clinical background and QOL. Oskar et al. described that 75% men diagnosed with prostate cancer reported positive lifestyle changes after diagnosis [12]. They also described that low-risk prostate cancer patients showed healthy lifestyle changes after diagnosis; the time after diagnosis may be a “teachable” moment that facilitates lifestyle interventions [13]. Thus, we used clinical background and QOL indicators as external criteria for criterion-related validity and concurrent validity of the HLCI.

Our results suggest more cases with high HLCI (T3) than with low HLCI (T1) during treatment. This is consistent with results suggesting that cancer diagnosis leads to healthy lifestyle changes. We also showed that T3 was most common in recurrent patients and at stage III/IV, indicating that poor cancer prognosis may be a more “teachable” experience than favorable prognosis.

The global health status of the EORTC QLQ-C30 indicates general QOL status because it comprises the following two items: (1) How would you rate your overall health during the past week? (2) How would you rate your overall QOL during the past week [10]? Our findings indicated that patients conscious of healthy lifestyles showed better QOL than patients not conscious of healthy lifestyle. Oskar et al. reported that low-risk prostate cancer patients who exercised more and were interested in relationships and social activities reported improved overall QOL, indicating that better QOL leads to a healthier lifestyle than poor QOL [13]. Breast cancer patients who felt fatigued had lower physical activity and nutrition scores than patients without fatigue, suggesting that poor QOL is associated with less healthy lifestyles [14]. However, it remains controversial whether high QOL status leads to improved consciousness regarding healthy lifestyle or whether cancer patients conscious of healthy lifestyle have improved QOL.

Hall et al. reported that higher fear of cancer recurrence causes more emotional distress and reduced health behaviors among cancer survivors [12]. Fear of cancer recurrence is also reported to be associated with increased physical symptoms, suggesting that physical and psychological symptoms are related [12]. Trudel-Fitzgerald et al. also reported that high anxiety and depression symptoms among women with colorectal cancer were associated with unhealthy lifestyles [15]. These findings are consistent with our results showing that gynecological cancer patients with severe depression are not extremely conscious of a healthy lifestyle.

Barriers to provision of lifestyle advice reported by healthcare providers include a perception that patients lack interest in behavioral change; a perception that behavioral change is not feasible or effective; and a potential risk of blaming the patient [16]. Healthcare professionals should provide lifestyle advice tailored to the individual [16]. There is no care for a healthy lifestyle that is universally applicable [16]. Therefore, the care for a healthy lifestyle of patients with cancer should be tailored to the individual according to their level of health consciousness. The HLCI helps stratify the level of health consciousness of patients with cancer, allowing for care for a healthy lifestyle to be tailored to the individual.

Further studies are necessary to improve and validate the HCLI. This study has several limitations that HLCI should be considered in future studies. First, the HLCI was not evaluated before and after treatment in the same patient. Longitudinal HLCI data should be collected on the same person to assess the changes in the HLCI before and after treatment in detail. Second, the utility of the HLCI for the precision care for a healthy lifestyle was not evaluated because the HLCI was not assessed according to the specific clinical backgrounds of patients with cancer. Further assessments should be stratified according to the level of health consciousness of the individual.

In conclusion, we developed a HLCI for gynecological cancer patients and confirmed its reliability and validity. This is the first index to assess patient awareness of a healthy lifestyle. HLCI can be used to assess consciousness of cancer patients regarding healthy lifestyle and in precision care for healthy lifestyle and improved QOL.
